# Mind the Gap: A Nationwide Survey on Diagnostic and Therapeutic Capacity for Invasive Fungal Infections in Chile

**DOI:** 10.3390/jof12100772

**Published:** 2026-10-15

**Authors:** Cinthia Carleo, Christian Conde-Abeliuk, Cecilia Tapia, Andrés Soto, Natalia Díaz, Mariella Raijmakers

**Affiliations:** 1 Hospital Alto Hospicio, Alto Hospicio 1130000, Chile; cinthiacarleoc@gmail.com; 2 Sociedad Chilena de Infectología, Santiago 8320000, Chile; christian.condemd@gmail.com (C.C.-A.); cecilia.tapiap@hoscar.cl (C.T.); andresoto@uchile.cl (A.S.); natalia.andreadh@gmail.com (N.D.); 3 Hospital Barros Luco Trudeau, Santiago 8900085, Chile; 4 Laboratorio Clínico, Hospital de Carabineros, Santiago 7770201, Chile; 5 Unidad de Infectología, Hospital del Salvador, Santiago 7500922, Chile; 6 Facultad de Medicina, Universidad de Chile, Santiago 8380453, Chile; 7 Fundación Arturo López Pérez, Santiago 7500921, Chile; 8 Hospital Clínico Dr. Félix Bulnes Cerda, Santiago 9080000, Chile

**Keywords:** invasive fungal infection, survey, antifungal access, therapeutic drug monitoring

## Abstract

**Background:** Invasive fungal infections can cause morbidity and mortality, particularly among immunocompromised and critically ill patients, making them an important global public health problem. Outcomes depend on timely diagnosis, laboratory capacity, and access to effective antifungal therapy. As national data describing these resources in Chile remain limited, we aimed to characterize national gaps in mycological diagnostic and therapeutic access. **Methods:** We conducted a nationwide survey of physicians, pharmacists, and laboratory professionals representing Chilean hospitals. Multiple responses from the same institution were consolidated. A 15-item diagnostic and therapeutic access score was developed to assess differences between hospitals. **Results:** Thirty-five hospitals participated in the survey. Diagnostic infrastructure varied substantially across institutions; all hospitals reported access to direct mycological examination, among which 65.7% had access to MALDI-TOF MS and 51.4% used molecular identification methods. The access score differed significantly according to hospital size, with median scores of 53.3% (20.0–80.0), 66.7% (46.7–86.7), and 73.3% (60.0–86.7) in hospitals with 0–300, 301–499, and ≥500 beds, respectively (*p* = 0.003). **Conclusions:** This nationwide assessment identified size-dependent inequities in mycological capacity in Chile, with larger hospitals having greater diagnostic and therapeutic access. These findings support targeted national strategies to strengthen smaller institutions and ensure equitable care.

## 1. Introduction

Fungal diseases have a broad clinical spectrum, ranging from non-invasive mucosal yeast infections to life-threatening invasive diseases caused by both yeasts and filamentous fungi [1,2]. In the last decade, the clinical relevance of these pathologies has increased due to the growing population of patients with advanced immunosuppression and the emergence of resistance to conventional antifungals [3,4].

A timely and accurate diagnosis is the most critical factor determining patient prognosis [5]. In Chile, recent multicenter data highlighted a significant clinical challenge: while therapeutic intervention rates are high among patients with suspected fungal diseases, long-term survival remains suboptimal [6]. This suggests a diagnostic–therapeutic gap where the availability of antifungal drugs does not necessarily translate into clinical success, potentially due to delays in species-level identification, limited access to specialized diagnostic tools, or insufficient implementation of therapeutic drug monitoring (TDM) and antifungal susceptibility testing [6,7].

The management of fungal diseases requires a robust, multidisciplinary infrastructure. According to the European Confederation for Medical Mycology (ECMM), excellent fungal care is built upon synergy between clinical expertise, specialized laboratory diagnostics—including MALDI-TOF-MS, antigen detection, and molecular methods—and clinical pharmacy through Antifungal Stewardship (AFS) programs [8,9]. However, the real-world impact of these resources is often limited by a lack of institutional awareness; if the multidisciplinary team is unaware of the diagnostic or therapeutic tools available in their own centers, patient care is inevitably compromised [8,10].

Although several studies in Latin America have mapped general laboratory infrastructure, no comprehensive assessment exists for Chile, and the degree of multidisciplinary coordination within Chilean hospitals remains unclear [11,12]. To address this, the Medical Mycology Advisory Committee of the Chilean Society of Infectious Diseases (SOCHINF) conducted a nationwide survey targeting clinicians, microbiologists, and pharmacists. This study was conducted to characterize the current landscape of fungal disease management in Chile, identify systemic barriers in diagnostic capacity and antifungal stewardship, and inform future health policies and institutional improvements.

## 2. Materials and Methods

A descriptive, cross-sectional, multicenter study was conducted between april 23th and june 5th 2026, using an electronic survey designed to characterize diagnostic and therapeutic access to antifungal resources in hospitals in Chile. Hospitals were selected as referral centers from each region of the country, comprising 46 institutions in total: 24 located in the Metropolitan Region, the capital of Chile, and 22 located outside the capital. The survey was developed in Google Forms and distributed to professionals involved in the management of fungal infections at each institution, including infectious disease physicians or other clinical physicians, pharmacists, and mycology laboratory professionals, such as medical technologists or microbiologists. The instrument consisted primarily of multiple-choice questions, together with selected free-text fields, aimed at collecting information on hospital characteristics, level of hospital complexity, patient population served, and the availability of a mycology laboratory, diagnostic methods, fungal identification, and antifungal susceptibility testing, as well as access to biomarkers, molecular assays, and therapeutic drug monitoring, and the availability of systemic antifungal agents. For this study, “availability” was defined as institutional access to a diagnostic method or antifungal agent whenever clinically required; occasional or intermittent access was not considered available. The survey was designed and refined by members of the Medical Mycology Advisory Committee. Its structure and content were adapted from comparable international studies evaluating mycology capacity and antifungal access [11,12]. The survey is available in Table S1.

For the analysis, individual responses were consolidated at the hospital level because more than one professional from some centers responded. Hospital names were standardized to identify responses corresponding to the same institution. For single-response variables, the most frequent response within each hospital was used. In contrast, for multiple-choice variables, the combination of the capabilities reported by the different professionals from the same center was used, interpreting the presence of capability as institutionally reported availability. Residual discrepancies between responses from the same hospital were also identified as potential indicators of heterogeneity in institutional knowledge. In the case of discrepancies, the information was confirmed with each center. Categorical variables were expressed as absolute frequencies and percentages, whereas continuous variables were summarized as medians and interquartile ranges (IQRs).

The analysis was descriptive at the hospital level for the main diagnostic, therapeutic, and monitoring variables. To evaluate differences between groups, comparisons were performed according to hospital complexity, number of beds, and geographic region of the country. Because of the small numbers of low- and medium-complexity hospitals, they were grouped into a single category for complexity comparisons. For the geographic analysis, hospitals were classified by national region as follows: central (capital of Chile), northern, or southern. In addition, the central region was compared with the combined northern/southern regions to explore the hypothesis of a greater concentration of resources in the central part of the country. Categorical variables were compared using Fisher’s exact test or chi-square test, as appropriate.

We also developed an exploratory antifungal diagnostic and therapeutic access index to characterize institutional capacity for the diagnosis and management of invasive fungal infections. The index was not adapted from a previously validated framework. Fifteen variables were selected based on their clinical relevance to the management of invasive fungal infections and their incorporation into diagnostic and therapeutic recommendations in international clinical guidelines. The components included an institutional mycology laboratory; mycological diagnosis always performed within the institution; direct mycological diagnosis; MALDI-TOF; identification PCR; susceptibility testing for yeasts; susceptibility testing for filamentous fungi; galactomannan; (1 → 3)-β-D-glucan; *Pneumocystis jirovecii* PCR; voriconazole plasma concentration monitoring; and the availability of voriconazole, echinocandins, liposomal amphotericin B, and isavuconazole. Components were equally weighted (1 point each) to descriptively assess the level of institutional access while avoiding subjective assumptions regarding their relative clinical importance. Given the exploratory nature of the index and the absence of an evidence-based weighting framework, alternative weighting approaches were not evaluated. The total score was expressed as a percentage of the maximum possible score and categorized as low (<40%), intermediate (40–69.9%), or high (≥70%). Overall scores were compared using nonparametric tests (Mann–Whitney for two-group comparisons and Kruskal–Wallis for comparisons among three groups), as appropriate. The index was not externally validated and was used for descriptive comparison of structural access gaps between hospitals. A *p*-value ≤0.05 was considered statistically significant. Statistical analyses were performed using Stata/SE version 19.5 (StataCorp LLC, College Station, TX, USA).

## 3. Results

**Figure 1:**
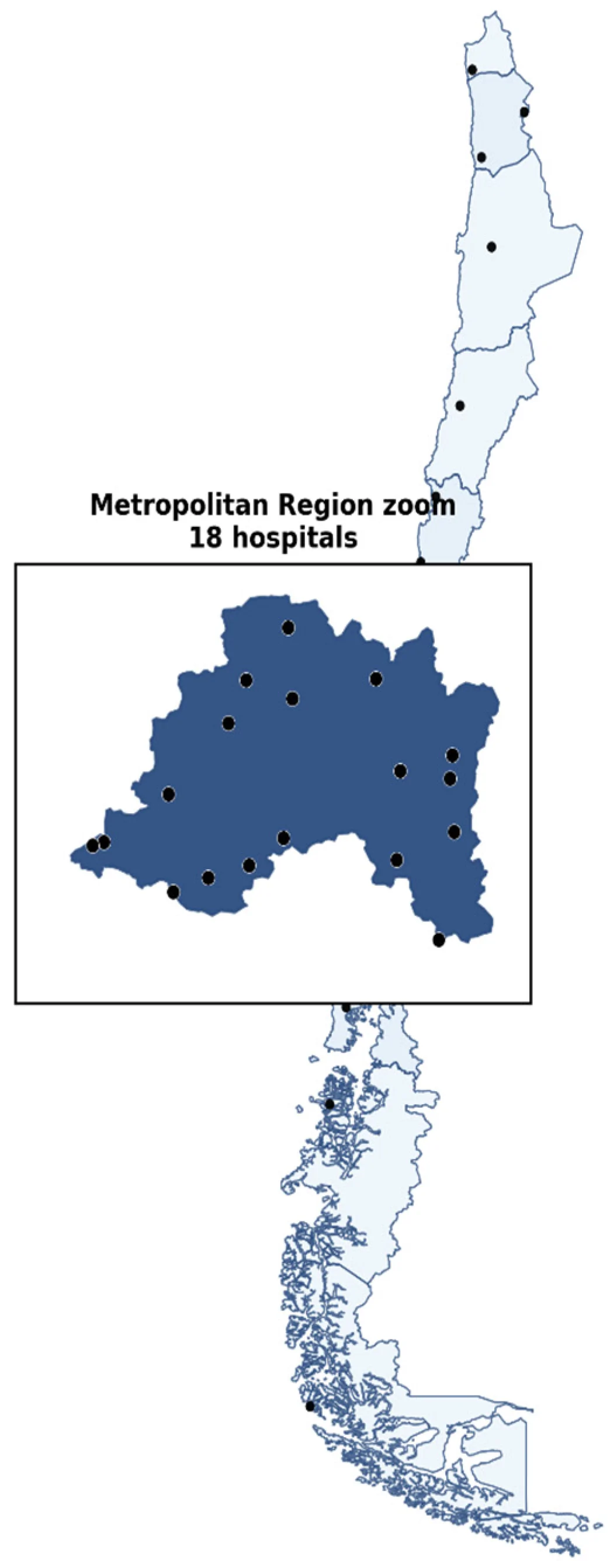
Geographic distribution of surveyed hospitals across Chile. Each black dot represents one participating hospital. The inset provides an enlarged view of the Metropolitan Region, where 18 participating hospitals were located.

A total of 35 of the 46 selected hospitals (76.1%) participated in the study, corresponding to 13 of the 16 regions of the country. Participation was based on 59 individual responses, which were consolidated at the institutional level. Of these, 30/35 hospitals (85.7%) were classified as high-complexity, 4 (11.4%) as intermediate-complexity, and 1 (2.9%) as low-complexity. In total, 30/35 hospitals (85.7%) reported providing care for patients considered at high risk for invasive fungal infection, operationally defined as centers caring for patients with hematologic malignancies, oncologic conditions, or transplantation. Of the participating hospitals, 18/35 (51.4%) were located in the Metropolitan Region, indicating that the capital had the highest concentration of participating centers (Figure 1). At the hospital level, there were responses from a single professional group for 19/35 (54.3%) hospitals, responses from two groups for 11/35 (31.4%) hospitals, and responses from all three groups for 5/35 (14.3%) hospitals. Physicians from 28/35 hospitals (80.0%), pharmacists from 19/35 (54.3%) hospitals, and laboratory/microbiology professionals from 9/35 (25.7%) hospitals responded. This distribution should be considered when interpreting technical variables related to mycological diagnosis, given that laboratory professionals were underrepresented relative to the other professional groups. The fungi most frequently reported as relevant or of greatest concern by the participating centers were *Candida* spp., which was mentioned by 33/35 hospitals (94.3%), followed by *Aspergillus* spp. in 24/35 hospitals (68.5%) and *Cryptococcus* spp. in 15/35 hospitals (42.9%). Less frequently reported fungi included Mucorales (17.1%) and *Histoplasma* spp. (5.7%) and dermatophytes (2.9%).

### 3.1. Diagnostic Tools

#### 3.1.1. Microscopy/Identification Tools

All centers have an institutional mycology laboratory. Direct mycological examination was widely available and was reported by all 35 hospitals (100%). The most frequently available microscopy-based methods were Gram staining (33/35, 94.3%); India ink staining (29/35, 82.9%); and potassium hydroxide (KOH) preparation (22/35, 62.9%). Other reported techniques included Giemsa staining (18/35, 51.4%), lactophenol cotton blue staining (16/35, 45.7%), calcofluor white staining (12/35, 34.3%), and silver staining (13/35, 37.1%) (Table 1).

Regarding culture medium availability, Sabouraud agar with or without an inhibitor was the most frequently available medium (27/35 hospitals; 77.1%), followed by chromogenic agar (16/35; 45.7%), and potato dextrose agar (11/35; 31.4%). Chromogenic agar was predominantly available in high-complexity centers, suggesting greater access to specialized mycological diagnostic resources in these institutions. Dermatophyte agar showed limited availability (2/35; 5.7%), while 7/35 centers (20.0%) did not know or did not report which culture media were available.

Fungal species identification by any method was widely available, being reported by 34/35 hospitals (97.1%). However, advanced fungal identification methods (PCR, MALDI-TOF MS, or automated VITEK-type systems) were available in only 26/35 hospitals (74.3%). Automated identification or VITEK-type systems were the most frequently available (26/35, 74.3%), followed by MALDI-TOF MS (23/35, 65.7%), PCR-based identification (18/35, 51.4%), and manual biochemical identification (13/35, 37.1%) systems. Combined access to MALDI-TOF MS and an automated VITEK-type system was reported by only 17/35 hospitals (48.6%), and the availability of all three advanced identification methods (PCR, MALDI-TOF MS, and VITEK-type systems) was limited to 11/35 hospitals (31.4%). Notably, all centers reporting access to more than one advanced identification method were high-complexity hospitals. Antifungal susceptibility testing for yeasts was widely available (29/35, 82.9%), whereas only 6/35 (17.1%) hospitals reported susceptibility testing for filamentous fungi. The same six centers reported availability of susceptibility testing for both yeasts and filamentous fungi. Regarding the methods used for antifungal susceptibility testing, automated systems (including VITEK) were the most frequently available (21/35, 60.0%), followed by broth microdilution (19/35, 54.2%), E-test (16/35, 45.7%), and disk diffusion (15/35, 42.9%).

#### 3.1.2. Antibody Detection Tests and Molecular Testing

Serologic antibody detection assays were uncommon, being available in 7 of 35 hospitals (20.0%). In contrast, antigen detection assays were available in 26 of 35 hospitals (74.3%). The most frequent were *Cryptococcus* spp. antigen and *Aspergillus* galactomannan, both of which were reported by 20 of 35 hospitals (57.1%). Access to (1 → 3)-β-D-glucan was limited, and it was available in only 5 of 35 centers (14.3%). Molecular tests were reported as available in 25 of 35 hospitals (71.4%). The most frequent was PCR for *Pneumocystis jirovecii*, which was available in 21 of 35 centers (60.0%), followed by PCR for *Candida* spp. in 11 of 35 (31.4%) and PCR for *Aspergillus* spp. in 4 of 35 (11.4%) hospitals. PCR for other fungi was reported by 4 of 35 hospitals (11.4%).

### 3.2. Therapeutic Access

**Figure 2:**
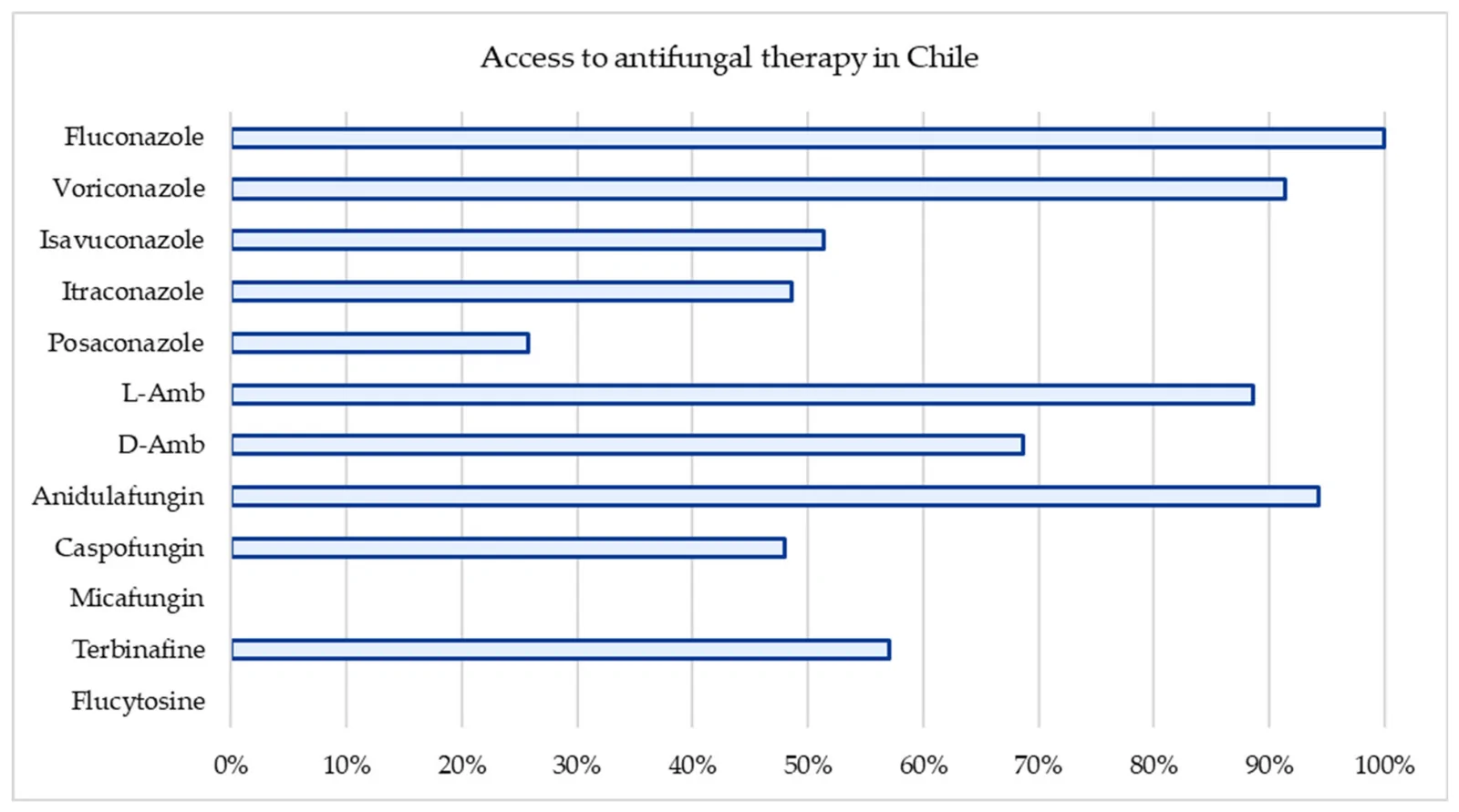
Access to antifungal therapy in Chile.

Regarding therapeutic access, systemic antifungal agents were broadly available across the participating centers. All hospitals reported access to at least one triazole, with fluconazole universally available (35/35, 100.0%), followed by voriconazole (32/35, 91.4%), isavuconazole (18/35, 51.4%), itraconazole (17/35, 48.6%), and posaconazole (9/35, 25.7%). Notably, access to isavuconazole demonstrated significant geographic heterogeneity, with higher availability among hospitals located in the Metropolitan Region, the country’s capital area (12/18, 66.7%), compared with hospitals from the northern and southern regions (6/17, 35.3%), suggesting an unequal distribution of access to newer-generation antifungals across the country. Availability of at least one echinocandin was reported by nearly all centers (34/35, 97.1%), mainly anidulafungin (33/35, 94.3%) followed by caspofungin (17/35, 48.6%). No center reported having micafungin available. Access to any formulation of amphotericin B was reported by 34/35 hospitals (97.1%), including liposomal amphotericin B (31/35, 88.6%) and amphotericin B deoxycholate (24/35, 68.6%). Terbinafine was available in 20/35 centers (57.1%), whereas no hospital reported the availability of flucytosine (Figure 2).

#### TDM

Access to antifungal therapeutic drug monitoring was limited. Overall, 20 of 35 hospitals (57.1%) reported access to plasma concentration monitoring for at least one antifungal agent. Monitoring was concentrated primarily on voriconazole, which was available in 18 of 35 hospitals (51.4%). In contrast, plasma concentration monitoring for isavuconazole and posaconazole was reported by only 2 of 35 (5.7%) and 1 of 35 (2.9%) hospitals, respectively.

### 3.3. Overall Access Score

The overall access score was compared according to hospital complexity, the geographic region of the country (central, northern, and southern regions), and the number of hospital beds (<300, 301–499, and ≥500 beds).

Thirty high-complexity hospitals and five medium-/low-complexity hospitals were analyzed. The overall score for access to antifungal diagnostic and therapeutic resources was higher in the high-complexity hospitals, with a median of 66.7% (IQR, 26.7–86.7%), compared with 60.0% (IQR, 20.0–80.0%) in the medium-complexity hospitals. However, this difference was not statistically significant (*p* = 0.272).

When high-complexity hospitals were compared with medium-/low-complexity hospitals, a trend toward greater availability of advanced diagnostic tools in high-complexity hospitals was observed. This difference was particularly evident for MALDI-TOF MS (73.3% vs. 20.0%; *p* = 0.038) and identification by polymerase chain reaction (PCR; 56.7% vs. 20.0%; *p* = 0.1774). However, most between-group differences did not reach statistical significance, possibly because of the small number of medium- or low-complexity hospitals included in the analysis.

**Figure 3:**
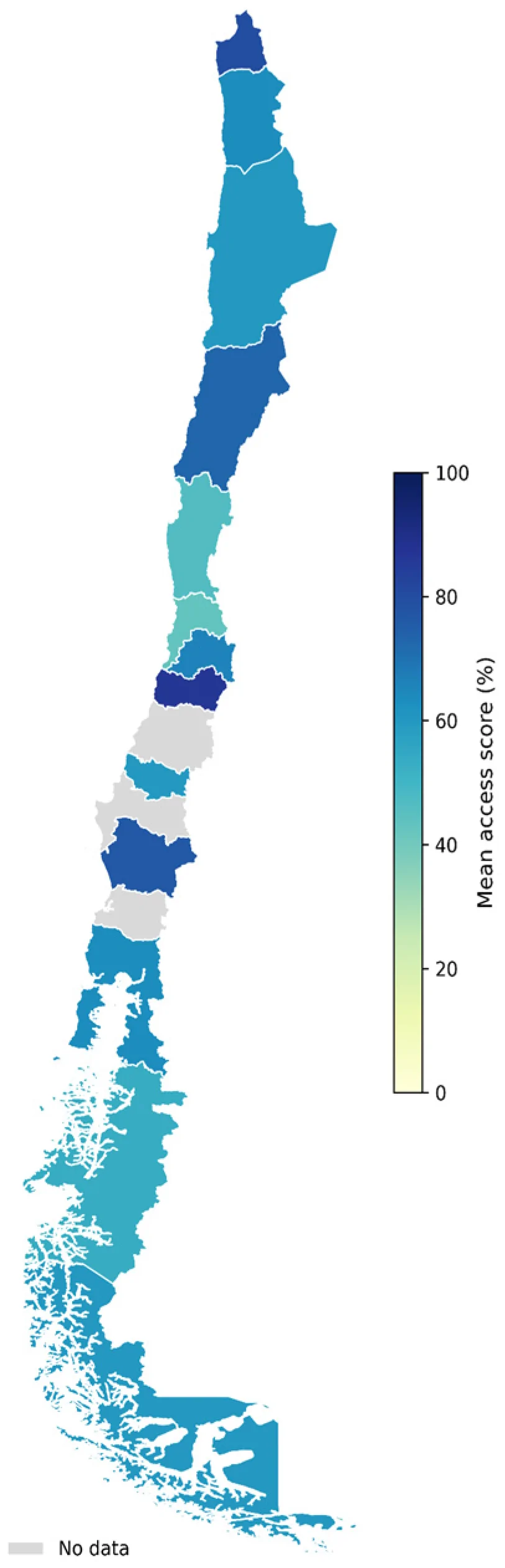
Distribution of the overall access score according to geographic region of the country. Regional scores are represented using a continuous yellow-to-blue color scale, with yellow indicating lower access and dark blue indicating higher access. Regions shown in gray had no available data.

Descriptively, the overall access score was highest in the central region, with a median of 73.3%, compared with the northern (60.0%) and the southern (63.3%) regions. No statistically significant differences were observed among the three regions (*p* = 0.432) (Supplementary Table S2, Figure 3). When hospitals in the central region were compared with those in the northern and southern regions combined, greater availability was observed for MALDI-TOF MS (14/18; 77.8 vs. 9/17; 52.9%; *p* = 0.164), voriconazole therapeutic drug monitoring (11/18 [61.1%] vs. 7/17 [41.2%]; *p* = 0.318), and isavuconazole (12/18 [66.7%] vs. 6/17 [35.3%]; *p* = 0.094). However, none of these differences reached statistical significance. Overall, these findings do not conclusively support the hypothesis of greater overall availability of antifungal resources in the central region.

**Figure 4:**
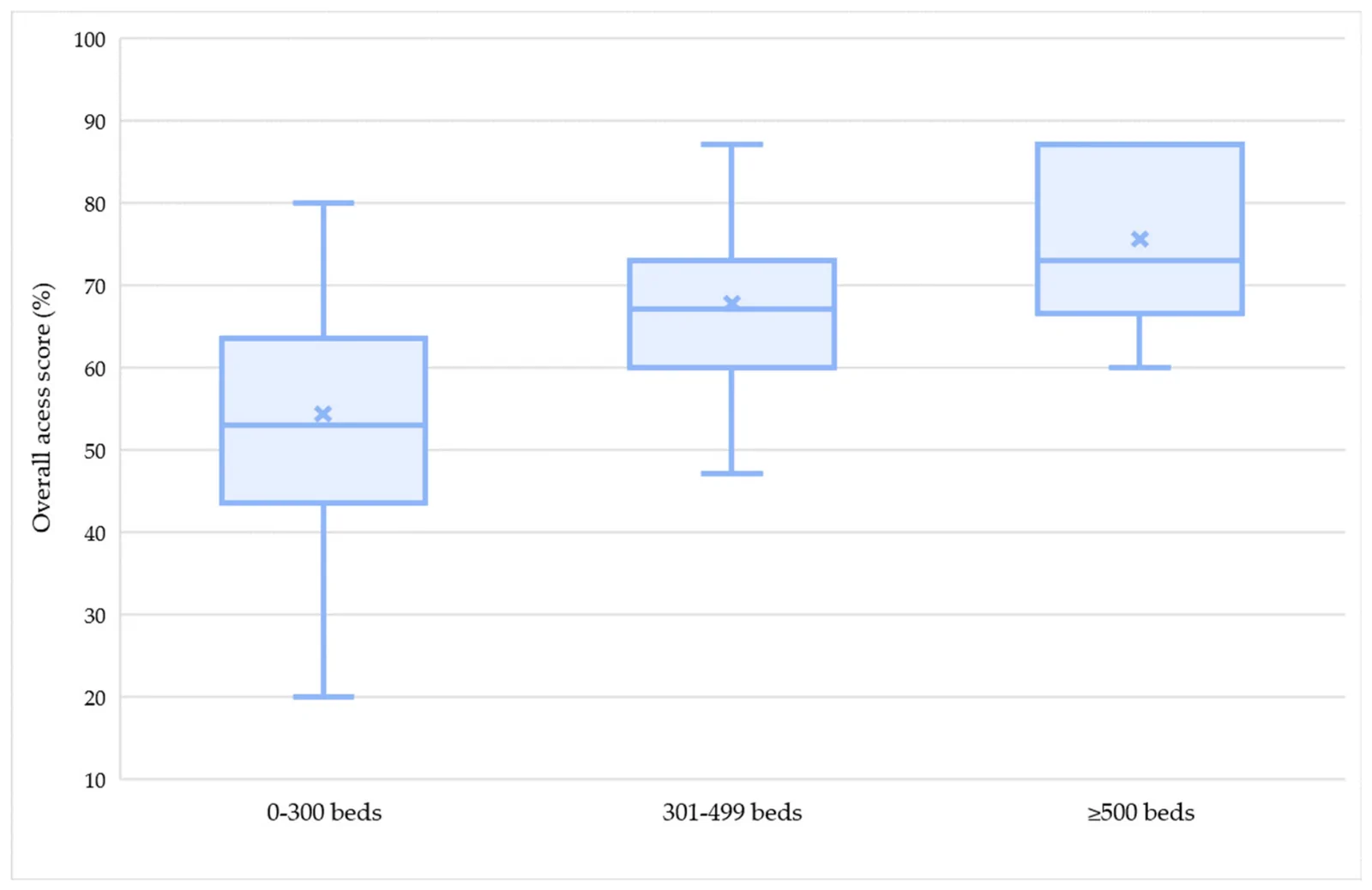
Box plot of overall access score according to hospital beds (<300, 301–499, and ≥500 beds).

A clear association was observed between hospital size and the overall access score. Hospitals with fewer than 300 beds had a median score of 53.3% (IQR, 20.0–80.0%), whereas hospitals with 301–499 beds had a median score of 66.7% (IQR, 46.7–86.7%), and those with 500 or more beds had a median score of 73.3% (IQR, 60.0–86.7%). The difference among the three groups was statistically significant according to the Kruskal–Wallis test (*p* = 0.003) (Figure 4).

MALDI-TOF MS showed the most pronounced between-group difference. Its availability increased progressively with the number of hospital beds from 30.8% in hospitals with fewer than 300 beds to 76.9% in hospitals with 301–499 beds and 100% in hospitals with 500 or more beds. This difference was statistically significant (*p* = 0.002).

Other indicators also showed a trend toward greater availability in larger hospitals, although the differences did not reach statistical significance. These indicators included the availability of voriconazole, posaconazole, isavuconazole, PCR identification, and *Cryptococcus* antigen testing (Table 2). These findings suggest that hospital size may be associated with greater access to advanced diagnostic technologies and specialized antifungal agents. However, some comparisons may have been limited by the sample size.

## 4. Discussion

In this nationwide survey, most participating hospitals in Chile reported caring for patient populations at high risk for invasive fungal infections (IFIs), predominantly individuals with hematologic malignancies, oncologic conditions, or those undergoing transplantation. This finding underscores the urgent need to strengthen diagnostic and therapeutic capacity for fungal diseases across the country.

The fungi most frequently identified as clinically relevant were *Candida* spp., *Aspergillus* spp., and *Cryptococcus* spp., an observation that closely aligns with global and regional epidemiology reported in recent years [13,14]. This study included hospitals from 13 of Chile’s 16 national regions, and a trend toward reduced diagnostic capacity in geographically extreme regions was observed, particularly regarding access to specialized mycology laboratories. However, these regional differences did not reach statistical significance.

Diagnostic infrastructure varied substantially across institutions. While all hospitals reported access to direct mycological examination, only 65.7% had MALDI-TOF MS, and 51.4% reported access to molecular identification methods. These advanced techniques were predominantly available in high-complexity hospitals, exposing structural inequities in diagnostic access. Antifungal susceptibility testing was widely available for yeasts (82.9%) but critically limited for filamentous fungi (17.1%), indicating that susceptibility testing for molds has not been fully integrated into AFS programs. The lack of standardization across susceptibility methods and the limited availability of therapeutic drug monitoring (TDM)—available for voriconazole in only 51.4% of centers and virtually absent for posaconazole (2.9%) and isavuconazole (5.7%)—further illustrate profound gaps in the diagnostic–therapeutic continuum.

In addition to these gaps, access to fungal antigen detection was heterogeneous: *Aspergillus* galactomannan was available in only 48.6% of hospitals (ELISA) and 20% (lateral flow assay), while *Cryptococcus* antigen testing—critical for the diagnosis of cryptococcal meningitis—was available in 34.3% of centers via lateral flow assay and 42.9% via latex agglutination. *Histoplasma* antigen detection was reported in only 8.6% of hospitals, underscoring substantial limitations in the diagnosis of endemic mycoses. This limited and uneven access to antigen-based diagnostics is clinically relevant because these assays provide rapid, non-culture-based detection of key fungal pathogens, enabling earlier therapeutic decisions and improving outcomes in settings where advanced identification methods or molecular testing are not consistently available.

Access to (1•3)-β-D-glucan (BDG) was notably scarce and reported by only 14.3% of hospitals. BDG is a cell wall polysaccharide used as a pan-fungal biomarker to support diagnosis and guide therapy in IFI. Its diagnostic performance varies according to patient population and pretest probability, offering a high negative predictive value (>90%) in critically ill patients and solid organ transplant recipients when the likelihood of IFI is low to moderate [15]. However, BDG interpretation remains challenging due to variability in assay performance across manufacturers, as highlighted in a 2020 Cochrane review [16]. Beyond invasive candidiasis and aspergillosis, BDG also plays a role in diagnostic algorithms for *Pneumocystis jirovecii* pneumonia (PJP). In a multicenter European ICU study, BDG demonstrated a sensitivity of 86% and specificity of 71%, with its positive predictive value improving when combined with respiratory PCR [17]. Importantly, BDG has also been shown to safely shorten antifungal therapy duration in invasive candidiasis, reducing unnecessary exposure to antifungals [18]. The limited availability of BDG in Chilean hospitals therefore represents a critical bottleneck in early, non-invasive diagnosis and stewardship.

Although most hospitals reported broad access to first-line systemic antifungal agents, important therapeutic gaps persist. Only 51.4% of centers had access to isavuconazole, an agent used alongside voriconazole for invasive aspergillosis and an approved therapy for mucormycosis. Furthermore, given that therapeutic drug monitoring for voriconazole was available in only 51.4% of participating centers, gaining access to isavuconazole—which generally does not require routine TDM—is critical. Access to isavuconazole also revealed marked geographical centralism, with 66.7% of centers in the Metropolitan Region having access compared to only 35.3% in the northern and southern regions. While international guidelines support its use as first-line therapy for invasive aspergillosis and as rescue or sequential therapy for mucormycosis—particularly when liposomal amphotericin B cannot be used due to toxicity—its limited availability outside the capital may restrict optimal management, especially in the absence of therapeutic drug monitoring in the blood of infected individuals [19,20,21].

Regarding other classes, while anidulafungin was universally available (94.3%), micafungin was completely absent across all centers. Most critically, access to flucytosine was absent in all surveyed hospitals (0%) despite its long-standing role in induction therapy for cryptococcal meningitis and its demonstrated impact on reducing mortality and hospitalization in resource-limited settings [22,23,24,25]. As a result, induction therapy for cryptococcal meningitis in Chile often relies on liposomal amphotericin B plus fluconazole.

Hospital size (number of beds) emerged as the most statistically significant determinant of diagnostic and therapeutic access (*p* = 0.003). Larger hospitals (≥500 beds) demonstrated significantly higher overall access scores (median 73.3%) and greater availability of advanced diagnostic tools, most notably MALDI-TOF MS (100% vs. 76.9% in hospitals 301–499 beds and 30.8% in hospitals <300 beds; *p* = 0.002), PCR-based identification, and specialized antifungal agents. This association suggests that structural capacity and institutional scale—rather than geographic region or hospital complexity alone—are the primary drivers of access to comprehensive IFI management resources in Chile.

This study has limitations that should be acknowledged. First, although the survey included hospitals across most national regions, it did not achieve complete coverage of all hospital centers in Chile. Second, the unequal representation of professional groups represents a methodological limitation—while physicians and pharmacists were well-represented (80.0% and 54.3%, respectively), only 25.7% of hospitals produced responses from laboratory professionals. Because information was gathered through self-reported survey data, this imbalance suggests potential underreporting of diagnostic capabilities and introduces information bias regarding diagnostic techniques. Conversely, self-reported availability may also overestimate actual institutional capacity, as reported access to a diagnostic test or an antifungal agent does not necessarily imply its consistent, timely, or routine availability in clinical practice. The survey did not independently verify the reported resources or assess their frequency of use, turnaround times, or potential operational barriers to access. Therefore, the results should be interpreted as reported institutional availability rather than confirmed real-world capacity. Finally, most participating institutions were high-complexity hospitals, and only a small number of medium- and low-complexity centers are represented, which may limit the generalizability of these findings across lower-tier healthcare settings in the country.

## 5. Conclusions

This nationwide survey revealed broad access to several core diagnostic and therapeutic resources for invasive fungal infections in Chile. Nevertheless, substantial gaps remain, particularly in access to BDG testing, antifungal susceptibility testing—especially for filamentous fungi—and therapeutic drug monitoring. Therapeutic gaps were also identified, most notably the absence of flucytosine, an essential component of recommended induction regimens for cryptococcal meningitis, and the limited availability of newer-generation antifungal agents. These findings provide a national baseline for developing public health policies to address current deficiencies, strengthen regional laboratory capacity, and ensure more timely and equitable access to fungal diagnostics and antifungal treatment across the country.

As part of this effort, the Medical Mycology Advisory Committee intends to implement targeted interventions to improve diagnostic and therapeutic access nationwide. Future initiatives will include educational programs and training courses to strengthen knowledge and awareness of available mycological diagnostic and therapeutic resources; advocacy efforts to facilitate the introduction of currently unavailable antifungal agents in Chile; and the development of centralized diagnostic strategies for specialized tests that may not be feasible to implement at every institution, such as (1 → 3)-β-D-glucan testing. Establishing a national reference laboratory for selected specialized mycological tests could be a practical approach to improving access, particularly for smaller hospitals.

## Figures and Tables

**Table 1:** Diagnostic, susceptibility, antigen, molecular, and therapeutic drug monitoring techniques.

Technique	Total (n)	%
Microscopy		
KOH/potassium hydroxide	22	62.9
Gram stain	33	94.3
India ink	29	82.9
Calcofluor white	12	34.3
Lactophenol cotton blue	16	45.7
Silver stain/Grocott stain in histology	13	37.1
Giemsa stain in histology	18	51.4
Cultures		
Chromogenic agar	16	45.7
Potato dextrose agar	11	31.4
Sabouraud agar with or without inhibitor	27	77.1
Dermatophyte agar	2	5.7
Unknown	7	20.0
Species identification		
Automated VITEK-type identification	26	74.3
Manual biochemical identification	13	37.1
MALDI-TOF-MS	23	65.7
PCR for identification	18	51.4
Microscopic/macroscopic identification	1	2.9
Unknown	2	5.7
Antifungal susceptibility testing technologies		
Disk diffusion	15	42.8
E-test	16	45.7
Broth microdilution	19	54.3
VITEK or other automated method	21	60
Unknown	3	8.6
Antibodies		
*Aspergillus* spp. antibodies	3	8.6
*Candida* spp. antibodies	3	8.6
*Cryptococcus* antibodies	1	2.9
*Histoplasma* spp. antibodies	2	5.7
Not available	28	80.0
Antigens		
*Aspergillus* GM (ELISA)	17	48.6
*Aspergillus* GM (LFA/immunochromatography)	7	20
(1 → 3)-β-D-glucan	5	14.3
*Cryptococcus* (LFA)	12	34.3
*Cryptococcus* (latex agglutination)	15	42.9
*Histoplasma*	3	8.6
Not available	8	22.9
Molecular		
*Aspergillus* PCR	4	11.4
*Candida* PCR	11	31.4
Mucorales PCR	1	2.9
*Pneumocystis jirovecii* PCR	21	60.0
*Cryptococcus* PCR in CSF	6	17.1
PCR for other fungi	5	14.3
FilmArray/molecular panel including fungi	2	5.7
No molecular techniques reported	10	28.6
Therapeutic drug monitoring		
Voriconazole TDM	18	51.4
Posaconazole TDM	1	2.9
Isavuconazole TDM	2	5.7
Not available	16	45.7

MALDI-TOF-MS: matrix-assisted laser desorption/ionization-time of flight mass spectrometry; PCR: polymerase chain reaction; E-test: Epsilometer test; GM: Galactomannan; LFA: Lateral Flow Assay; CSF: cerebrospinal fluid; TDM: Therapeutic Drug Monitoring.

**Table 2:** Individual Components of the Diagnostic and Therapeutic Access Score by Institutional Bed Capacity.

Item	0–300 Beds	301–499 Beds	≥500 Beds	*p*-Value
Institutional mycology laboratory	13/13 (100%)	13/13 (100%)	9/9 (100.0%)	
Always internal diagnosis	3/13 (23.1%)	5/13 (38.5%)	5/9 (55.6%)	0.374
Direct mycological diagnosis	13/13 (100.0%)	13/13 (100.0%)	9/9 (100.0%)	NA
MALDI-TOF-MS	4/13 (30.8%)	10/13 (76.9%)	9/9 (100.0%)	0.002
PCR identification	4/13 (30.8%)	8/13 (61.5%)	6/9 (66.7%)	0.688
Yeast susceptibility	9/13 (69.2%)	12/13 (92.3%)	8/9 (88.9%)	0.348
Filamentous susceptibility	1/13 (7.7%)	2/13 (15.4%)	3/9 (33.3%)	0.453
Galactomannan	6/13 (46.2%)	7/13 (53.8%)	7/9 (77.8%)	0.384
(1 → 3)-β-D-glucan	2/13 (15.4%)	2/13 (15.4%)	1/9 (11.1%)	1.0
*Pneumocystis jirovecii* PCR	6/13 (46.2%)	9/13 (69.2%)	6/9 (66.7%)	0.471
TDM voriconazole	6/13 (46.2%)	6/13 (46.2%)	6/9 (66.7%)	0.632
Voriconazole	10/13 (76.9%)	13/13 (100.0%)	9/9 (100.0%)	0.100
Equinocandin	12/13 (92.3%)	13/13 (100.0%)	9/9 (100.0%)	1.0
Liposomal amphotericin	10/13 (76.9%)	13/13 (100.0%)	8/9 (88.9%)	0.170
Isavuconazole	5/13 (38.5%)	6/13 (46.2%)	7/9 (77.8%)	0.225

## Data Availability

All data generated or analyzed in this study are included in the article and its Supplementary Materials. If additional information is required, it may be requested from the corresponding author.

## Supplementary Materials

The following supporting information can be downloaded at: https://www.mdpi.com/article/10.3390/jof12100772/s1, Table S1: Summary of the survey. Table S2: Individual Components of the Diagnostic and Therapeutic Access Score by Institutional Bed Capacity and Geographic Region. Table S3: Overall access score by geographic region.
