# Supplementary material for: Mind the Gap: A Nationwide Survey on Diagnostic and Therapeutic Capacity for Invasive Fungal Infections in Chile
Source: J Fungi (Basel). 2026 Oct 15;12(10):772. doi: 10.3390/jof12100772 (PMC13705827; doi:10.3390/jof12100772)
Supplement: Supplementary file 1 [file jof-12-00772-s001.zip › jof-4538540-supplementary.pdf]

Mind the Gap: A Nationwide Survey on Diagnostic and Therapeutic Capacity for Invasive Fungal Infections in Chile.

Supplementary Table S1: Summary of the survey.

1. **Institution profile**

1.1. Contact information

1.1.1. Email

1.2. Position

1.2.1. Attending Physician
1.2.2. Microbiologist
1.2.3. Director
1.2.4. Laboratory Professional
1.2.5. Internal Medicine Physician
1.2.6. Pharmacist
1.2.7. Pediatrician
1.2.8. Medical Technologist
1.2.9. Other: ____________________

1.3. Number of beds of the institution

1.3.1. 0–50
1.3.2. 50–100
1.3.3. 100–200
1.3.4. 200–300
1.3.5. 300–400
1.3.6. 400–500
1.3.7. 500–600
1.3.8. 600
1.3.9. I do not know

1.4. Name of the institution

1.4.1. Free answer

1.5. Facility complexity level

1.5.1. High-complexity hospital
1.5.2. Medium-complexity hospital
1.5.3. Low-complexity hospital

1.6. Does your hospital provide services for patients with any of the following conditions? (You may select more than one answer.)

1.6.1. COVID-19
1.6.2. Hematology-oncology patients
1.6.3. HIV/AIDS
1.6.4. Oncology
1.6.5. Solid organ transplantation
1.6.6. Hematopoietic stem cell transplantation
1.6.7. Severe burns unit

1.7. Which population is treated at your facility? *

1.7.1. Children
1.7.2. Adults
1.7.3. Both children and adults

1.8. Does your facility have access to a mycology laboratory?

1.8.1. Yes, within the institution
1.8.2. Yes, outside the institution

1.9. Where is the mycological diagnosis performed?

1.9.1. Always within the institution
1.9.2. Partly within the institution and partly outside the institution
1.9.3. All samples are referred to an external center
1.9.4. I do not know where the samples are processed
1.9.5. No access to ordering mycological tests

1.10. Which fungi are of greatest importance at your institution? (You may select more than one answer.)

1.10.1. *Aspergillus* spp.
1.10.2. *Candida* spp.
1.10.3. *Cryptococcus* spp.
1.10.4. *Fusarium* spp.
1.10.5. *Histoplasma* spp.
1.10.6. Mucorales

1.11. If a mycology laboratory is available, how many samples are processed per month?

1.11.1. Unknown
1.11.2. Free answer

1.12. Select antifungal agents available at your institution (You may select more than one answer.)

1.12.1. Anidulafungin
1.12.2. Caspofungin
1.12.3. Micafungin
1.12.4. Fluconazole
1.12.5. Itraconazole
1.12.6. Voriconazole
1.12.7. Isavuconazole
1.12.8. Posaconazole
1.12.9. Amphotericin B deoxycholate
1.12.10. Amphotericin B lipid complex
1.12.11. Liposomal amphotericin B
1.12.12. Other amphotericin B formulations
1.12.13. Flucytosine (5-FC)
1.12.14. Terbinafine

1.13. Which antifungal agent would you like to have available at your institution?

1.13.1. Free answer

1.14. **Microscopy: available methods. (You may select more than one answer.)**

1.14.1. India ink

1.14.2. KOH (potassium hydroxide)
1.14.3. Silver stain (histology)
1.14.4. Gram stain
1.14.5. Lactophenol blue
1.14.6. I do not know

1.15. Culture: available methods (You may select more than one answer.)
1.15.1. Chromogenic agar
1.15.2. Potato dextrose agar
1.15.3. Sabouraud agar (with or without inhibitor)
1.15.4. I do not know

1.16. Species identification: available methods. (You may select more than one answer.)
1.16.1. Automated identification (e.g., VITEK)
1.16.2. Manual biochemical identification
1.16.3. MALDI-TOF MS
1.16.4. PCR
1.16.5. I do not know
1.16.6. Antifungal susceptibility testing
1.16.7. Available for yeasts
1.16.8. Available for molds
1.16.9. Available for both
1.16.10. I do not know
1.16.11. None

1.17. Technologies. (You may select more than one answer.)
1.17.1. Broth microdilution
1.17.2. Etest
1.17.3. VITEK (or another system)
1.17.4. Disk diffusion
1.17.5. None
1.17.6. I do not know

1.18. Maximum identification capability for yeasts
1.18.1. Genus
1.18.2. Genus/species

1.19. Maximum identification capability for filamentous fungi.
1.19.1. Genus
1.19.2. Genus/species

1.20. **Serology (You may select more than one answer.)**
1.20.1. *Aspergillus* spp.
1.20.2. *Candida* spp.
1.20.3. *Histoplasma* spp.
1.20.4. None

1.21. Antigen detection (You may select more than one answer.)
1.21.1. *Aspergillus* galactomannan (ELISA)
1.21.2. *Aspergillus* galactomannan (LFA or immunochromatography)
1.21.3. *Candida* antigen

1.21.4. *Cryptococcus* (LFA)
1.21.5. *Cryptococcus* (latex agglutination)
1.21.6. *Histoplasma*
1.21.7. Beta-D-glucan
1.21.8. None

1.22. Molecular tests (You may select more than one answer.)

1.22.1. *Aspergillus* PCR
1.22.2. *Candida* PCR
1.22.3. *Pneumocystis* PCR
1.22.4. Mucorales PCR
1.22.5. PCR for other fungi
1.22.6. None

1.23. Do you have access to plasma-level monitoring for any of the following antifungal agents? (You may select more than one answer.)

1.23.1. Voriconazole
1.23.2. Posaconazole
1.23.3. Isavuconazole
1.23.4. None
1.23.5. Other: ____________________

For this study, "availability" was defined as institutional access to a diagnostic method or antifungal agent whenever clinically required; occasional or intermittent access was not considered available.

Supplementary Table S2. Individual Components of the Diagnostic and Therapeutic Access Score by Institutional Bed Capacity and Geographic Region.

**Institution**	**N beds**	**Region**	**IML**	**AID**	**DMD**	**MALDI-TOF**	**PCRi**	**YS**	**FS**	**GM**	**BDG**	**PCR Pn**	**TDM VCZ**	**VCZ**	**EQN**	**LAmB**	**ISA**	**Score**	**Category**
Hospital Alto Hospicio	0-300	North	1	0	1	0	0	1	0	1	1	0	0	1	1	1	1	9	Intermediate
Hospital Dr Exequiel Gonzalez Cortes	0-300	Central	1	0	1	1	0	1	0	0	0	0	1	1	1	1	1	9	Intermediate
Hospital Dr Lucio Cordova	0-300	Central	1	1	1	1	0	1	0	1	0	1	1	1	1	1	1	12	High
Hospital El Pino	0-300	Central	1	0	1	1	1	1	0	0	0	0	1	1	1	0	0	8	Intermediate
Hospital FACH	0-300	Central	1	0	1	0	0	0	0	1	1	0	1	1	1	1	1	9	Intermediate
Hospital Hanga Roa	0-300	North	1	1	1	0	0	0	0	0	0	0	0	0	0	0	0	3	Low
Hospital Regional de Coyhaique	0-300	South	1	0	1	0	1	1	0	0	0	1	0	1	1	1	0	8	Intermediate
Hospital Roberto del Rio	0-300	Central	1	0	1	0	0	1	1	0	0	0	0	1	1	1	0	7	Intermediate
Hospital San José de Melipilla	0-300	Central	1	0	1	0	1	1	0	0	0	1	0	0	1	0	0	6	Low
Hospital Villarrica	0-300	South	1	0	1	0	1	1	0	1	0	1	0	1	1	1	1	10	Intermediate
Hospital de Carabineros	0-300	Central	1	0	1	1	1	1	0	1	0	1	1	1	1	1	0	11	High
Hospital de Coquimbo	0-300	North	1	0	1	0	1	0	0	0	0	1	0	0	1	1	0	6	Intermediate
Hospital de La Serena	0-300	North	1	1	1	0	0	0	0	1	0	0	1	1	1	1	0	8	Intermediate
DIPRECA	300-500	Central	1	0	1	1	1	1	0	0	0	0	0	1	1	1	1	9	Intermediate
Hospital Carlos Van Buren	300-500	North	1	0	1	1	1	0	0	1	0	1	1	1	1	1	0	10	Intermediate
Hospital Clínico Herinda Martin Chillán	300-500	South	1	0	1	1	0	1	0	0	0	1	1	1	1	1	0	9	Intermediate
Hospital Clínico de Magallanes	300-500	South	1	1	1	0	1	1	0	0	0	1	0	1	1	1	0	9	Intermediate
Hospital Dr. Ernesto Torres Galdames	300-500	North	1	0	1	1	1	1	0	0	0	1	0	1	1	1	1	10	Intermediate
Hospital Eloísa Díaz de La Florida	300-500	Central	1	0	1	1	1	1	0	1	0	0	0	1	1	1	1	10	Intermediate
Hospital Luis Calvo Mackenna	300-500	Central	1	1	1	1	0	1	0	1	0	0	1	1	1	1	1	11	High
Hospital Militar de Santiago	300-500	Central	1	0	1	1	0	1	0	1	1	1	0	1	1	1	1	11	High
Hospital Padre Hurtado	300-500	Central	1	0	1	0	0	1	0	0	0	0	1	1	1	1	0	7	Intermediate
Hospital Regional de Arica Juan Noé Crevanie	300-500	North	1	1	1	0	1	1	0	1	1	1	1	1	1	1	0	12	High
Hospital Regional de Copiapo	300-500	North	1	1	1	1	1	1	1	0	0	1	0	1	1	1	0	11	High
Hospital de Osorno	300-500	South	1	1	1	1	0	1	0	1	0	1	0	1	1	1	0	10	Intermediate

Hospital del Salvador	300-500	Central	1	0	1	1	1	1	1	1	0	1	1	1	1	1	1	13	High
Hospital Barros Luco Trudeau	≥500	Central	1	1	1	1	0	1	0	1	0	1	1	1	1	1	1	12	High
Hospital Clínico Dr. Félix Bulnes Cerda	≥500	Central	1	0	1	1	1	1	1	1	0	1	0	1	1	1	0	11	High
Hospital Clínico San Borja Arriarán	≥500	Central	1	1	1	1	0	1	1	1	0	1	1	1	1	1	1	13	High
Hospital Dr. Hernán Henríquez Aravena	≥500	South	1	1	1	1	1	1	1	1	0	0	1	1	1	1	1	13	High
Hospital Dr. Sótero del Río	≥500	Central	1	1	1	1	1	1	0	1	0	0	0	1	1	1	1	11	High
Hospital Franco Ravera Zunino	≥500	South	1	0	1	1	1	1	0	1	1	1	1	1	1	1	1	13	High
Hospital Puerto Montt	≥500	South	1	0	1	1	1	1	0	0	0	1	1	1	1	0	0	9	Intermediate
Hospital Regional Antofagasta	≥500	North	1	0	1	1	1	0	0	0	0	1	0	1	1	1	1	9	Intermediate
Hospital San Juan de Dios de Santiago	≥500	Central	1	1	1	1	0	1	0	1	0	0	1	1	1	1	1	11	High

N, number; IML, institutional mycology laboratory; AID, Always internal diagnosis; DMD, Direct mycological diagnosis ; MALDI-TOF-MS, matrix-assisted laser desorption/ionization-time of flight mass spectrometry; PCRi, polymerase chain reaction for identification; YS, susceptibility testing for yeasts; FS, susceptibility testing for filamentous fungi; GM, galactomannan; BDG, Beta-D-glucan; PCR Pn; polymerase chain reaction for *Pneumocystis jirovecii* detection; TDM VCZ, Therapeutic drug monitoring of Voriconazole; VCZ, Voriconazole; LAmB, Liposomal amphotericin B; ISA, Isavuconazole, 1, available; 0, non- available.

Supplementary Table S3. Overall access score by geographic region.

**Region**	**N**	**Medium score (IQR)**	**p-value**
Central	18	73.3 (60.0–73.3)	0.432*
North	9	60.0 (53.3–66.7)	
South	8	63.4 (60.0–76.7)	

*Kruskal-Wallis
